# Satisfaction with academic supervision among nursing postgraduate students in China: a national multicenter cross-sectional study

**DOI:** 10.1186/s12909-026-09737-z

**Published:** 2026-06-24

**Authors:** Jiaqian Song, Liuyi Zhang, Xingzi Chen, Hanjing Wen

**Affiliations:** 1 School of Nursing, Hunan Normal University, Changsha, Hunan 410013 China; 2 The First Affiliated Hospital of Hunan Traditional Chinese Medical College (Hunan Provincial Directly Affiliated Hospital of Traditional Chinese Medicine), Zhuzhou, Hunan 412000 China

**Keywords:** Nursing education, Postgraduate students, Academic supervision, Supervisor, Satisfaction

## Abstract

**Background:**

High-quality academic supervision is essential to postgraduate nursing education, yet student-reported evidence on supervision quality remains limited, particularly across multiple institutions. This study examined the level of satisfaction with academic supervision among nursing postgraduate students in China and explored factors associated with higher satisfaction.

**Methods:**

A national multicenter cross-sectional survey was conducted among nursing postgraduate students from 30 universities in China between 2 April and 30 April 2024. Participants completed an anonymous online questionnaire assessing student and supervisor characteristics, team/mentorship structure, academic communication patterns, supervisor-student relationship classification, and learning motivation. Overall satisfaction with academic supervision was measured using a single-item 4-point ordinal scale. Ordinal logistic regression within the generalized linear model framework was used to identify factors associated with satisfaction with academic supervision.

**Results:**

Nursing postgraduate students generally reported high levels of satisfaction with academic supervision. Higher satisfaction was associated with challenge-oriented learning motivation, pursuit of money or other tangible incentives, positive team atmosphere and support from senior members, regular group meetings and knowledge sharing, guidance from multiple mentors, and “good teacher/helpful friend” and “task-oriented” supervisor-student relationships.

**Conclusion:**

Nursing postgraduate students generally reported high levels of satisfaction with academic supervision. Strengthening supervision structures and fostering supportive academic environments may further enhance academic supervision experiences in nursing postgraduate education.

## Background

 Building an advanced nursing workforce that supports population health and care quality requires postgraduate nursing education [1, 2]. However, growing postgraduate enrollment and a global scarcity of nursing supervisors have limited training capacity and raised supervision workloads [3], raising concerns about educational quality [4].

Quality assurance approaches vary internationally. In many high-income systems, such as the United States, the United Kingdom, and France, student-reported indicators are routinely combined with postgraduate outcomes to appraise program quality more holistically [5]. By contrast, Japan and South Korea rely more on agency-led, outcome-focused evaluations—emphasizing publications and employment—with comparatively limited student feedback [6–8]. In China, assessments are largely government-led and have historically under-weighted the student voice [9, 10], although recent policy calls for supervisor evaluation mechanisms that reasonably incorporate postgraduate feedback [11]. Supervisors play a central role in postgraduate training by providing academic guidance, feedback, and developmental support through academic supervision [12]. Students’ satisfaction with academic supervision may therefore serve as a student-centered indicator of the supervision experience and complement outcome-based indicators of postgraduate education quality [13].

Satisfaction with academic supervision refers to postgraduate students’ overall subjective evaluation of the quality of the academic supervision they receive during their training, particularly with respect to communication, availability, feedback, support, and opportunities for academic and professional development [14, 15]. Used as a student-centered, process-oriented indicator, it complements objective outputs (e.g., publications, completion, employment) and aligns with higher-education quality frameworks that integrate student experience indices [16, 17].

This study was guided by an Input–Environment–Outcome framework in higher education [18]. Within this framework, students’ demographic and academic characteristics and learning motivation were conceptualized as input factors, while supervision-related experiences, including communication with supervisors, supervisor–student relationships, team climate, group meetings, and guidance from multiple mentors, were conceptualized as environmental and process-related factors. Satisfaction with academic supervision was considered a student-reported outcome reflecting students’ overall evaluation of the supervision they received. This framework provided a rationale for examining how individual motivational factors and supervision structures may be associated with postgraduate nursing students’ satisfaction with academic supervision.

Despite the growing emphasis on postgraduate education quality, empirical evidence on nursing postgraduate students’ satisfaction with academic supervision remains limited. Existing quality assurance practices have tended to prioritize institutional or outcome-based indicators, while students’ subjective experiences of supervision have received comparatively less attention [10]. Moreover, the associations of student characteristics, supervisor-related factors, learning motivation, academic communication patterns, team/mentorship structure, and supervisor–student relationship with satisfaction with academic supervision remain insufficiently examined. Therefore, this study aimed to examine the level of satisfaction with academic supervision and its associated factors among Chinese nursing postgraduate students.

## Operational definitions

In this study, nursing postgraduate students referred to students formally enrolled in postgraduate nursing education programs in China, including both master’s and doctoral students, as well as both full-time and part-time students.

Satisfaction with academic supervision referred to students’ overall subjective evaluation of their supervision experience.

## Methods

### Study design

This was a national multicenter cross-sectional study.

### Settings

A national multicenter cross-sectional survey was conducted among nursing postgraduate students from 30 universities in China between 2 and 30 April 2024. A multistage stratified proportional cluster sampling strategy with voluntary recruitment within clusters was used. Universities were stratified by geographic region, including eastern, central, and western China, and 30 universities were selected in a 3:2:1 ratio according to the distribution of educational resources [19]. Each selected university was treated as a cluster. Within each cluster, designated institutional representatives and postgraduate schools distributed the online questionnaire link through commonly used student communication channels. Eligible students participated voluntarily after providing electronic informed consent. Therefore, individual-level random sampling was not performed within each university. Responses were anonymous, and the survey platform (www.wjx.cn) was configured to allow only one submission per device/IP.

### Participants and sample size

Students were included in the study if they (1) were nursing postgraduate students who were studying either full-time or part-time; (2) were Chinese citizens. Participants were excluded if they self-reported any of the following: (1) a severe physical or mental illness that could compromise their ability to provide informed consent or complete the questionnaire reliably; (2) current pregnancy, breastfeeding, or postpartum leave during the survey period [20]; or (3) use of treatments with well-documented potential short-term psychological or mood-related effects during the survey period, including short-term systemic corticosteroid regimens or gonadotropin-releasing hormone (GnRH) analogue treatment [21, 22]. These exclusion criteria were specified a priori to reduce the potential influence of transient physiological, hormonal, psychological, or leave-related factors on participants’ self-reported supervision experiences and satisfaction.

We calculated the sample size for a single proportion using $n=\frac{{z}_{\sigma}^{2}\times\:P\left(1-P\right)}{{d}^{2}}$ [23], with $\:{z}_{\sigma\:}$=1.96 (two-sided α = 0.05), prior Chinese surveys reported satisfaction with academic supervision of 88.90% [24] and 89.20% [25], respectively; we used the mean *p* = 0.8905 and an absolute margin of error d=0.03. This yielded *n*=417. Allowing a single 20% inflation for anticipated non-response/data loss in a multi-site online survey, the target sample was *n*=500.

In total, the questionnaire link was sent to 1043 nursing postgraduate students and 826 questionnaires were collected with a response rate of 79.19%. Excluding invalid questionnaires (regular responses), we obtained 814 valid questionnaires with an effectiveness rate of 98.55%.

### Instruments

The questionnaire was written in Chinese and included four sections. The survey used in this study was a composite questionnaire rather than a single previously published instrument. It consisted of study-specific single questions used to collect participant characteristics and supervision-related experiences, a supervisor-student relationship classification item based on a published classification, the Chinese version of the Working Preference Inventory, and a single-item measure of satisfaction with academic supervision.

#### General information questionnaire

These items were developed according to the study objectives and relevant literature on postgraduate supervision and educational experience, and were reviewed by the research team for content relevance, clarity, and appropriateness for nursing postgraduate students. They were intended to collect factual or contextual information rather than to form a psychometric scale; therefore, no total score was calculated, and internal consistency reliability, such as Cronbach’s alpha, was not applicable. The items covered four domains: (1) demographic characteristics: gender, marital status, degree format, degree program, and year of study; (2) academic background: undergraduate research experience; (3) supervisor characteristics: supervisor’s academic title, supervisor type, and supervisor’s project type; (4) supervision-related experiences: positive team atmosphere and support from senior members, regular group meetings and knowledge sharing, guidance from multiple mentors, and communicate candidly with supervisors to deepen mutual understanding, monthly academic communication with supervisors, weekly one-on-one communication time with supervisors, and primary mode of communication with supervisor. Positive team atmosphere and support from senior members, regular group meetings and knowledge sharing, guidance from multiple mentors, and communicate candidly with supervisors to deepen mutual understanding were recorded as dichotomous variables. Monthly academic communication with supervisors and weekly one-on-one communication time with supervisors were recorded as ordered categorical variables, as they were classified by communication frequency and duration, respectively. Primary mode of communication with supervisors was recorded as a nominal categorical variable, as it was classified by communication modality. The corresponding variables are presented in (Table 1).

**Table 1:** Participant characteristics and univariable comparisons of satisfaction with academic supervision (*N* = 814)

Variable	*n*(%)	Average rank	Statistic Z/H	*p*
Gender				
Male	163(20.00)	383.78	-1.71	0.087
Female	651(80.00)	413.44		
Marital status				
Married with children	70(8.60)	446.63	4.16	0.244
Married without children	58(7.13)	377.02		
Unmarried with partner	336(41.28)	406.72		
Unmarried without partner	350(43.00)	405.47		
Degree format				
Full-time	737(90.54)	406.29	-0.54	0.590
part-time	77(9.46)	419.04		
Degree program				
Professional degree	530(65.11)	410.50	-0.59	0.554
Academic degree	284(34.89)	401.89		
Year of study				
First-year master’s	354(43.49)	419.07	4.21	0.239
Second-year master’s	206(25.31)	398.70		
Third-year master’s	204(25.06)	407.33		
Doctoral	50(6.14)	362.50		
Undergraduate research experience				
Entrepreneurship	693(85.14)	413.75	-2.16	0.031
	121(14.86)	371.68		
Patent	722(88.70)	410.38	-1.17	0.244
	92(11.30)	384.89		
Writing project proposals	723(88.82)	406.12	-0.56	0.575
	91(11.18)	418.46		
Co-author papers	761(93.49)	410.11	-1.43	0.154
	53(6.51)	370.09		
First-author papers	654(80.34)	408.85	-0.39	0.694
	160(19.66)	401.99		
Authorship of books	689(84.64)	410.72	-1.09	0.275
	125(15.36)	389.73		
Intervention studies	692(85.01)	410.34	-0.98	0.328
	122(14.99)	391.37		
Cross-sectional studies	530(65.11)	401.68	-1.15	0.251
	284(34.89)	418.37		
Supervisor’s academic title				
Lecturer	65(7.99)	380.22	13.05	0.005
Associate professor	288(35.38)	397.88		
Professor	348(42.75)	433.93		
Other	113(13.88)	366.33		
Supervisor type				
Master’s supervisor	170(20.88)	407.61	-0.01	0.993
	644(79.12)	407.47		
Doctoral supervisor	568(69.78)	410.28	-0.61	0.542
	246(30.22)	401.09		
Fellow of the American Academy of Nursing	755(92.75)	411.41	-2.02	0.044
	59(7.25)	357.53		
Supervisor’s project type				
National-level projects	561(68.92)	400.51	-1.50	0.133
	253(31.08)	423.00		
Provincial-level projects	413(50.74)	402.51	-0.73	0.465
	401(49.26)	412.64		
Other projects	467(57.37)	415.99	-1.42	0.155
	347(42.63)	396.07		
No projects	715(87.84)	412.71	-2.02	0.043
	99(12.16)	369.84		
Positive team atmosphere and support from senior members				
No	728(89.43)	412.69	-2.18	0.029
Yes	86(10.57)	363.60		
Regular group meetings and knowledge sharing				
No	186(22.85)	294.88	-8.85	< 0.001
Yes	628(77.15)	440.86		
Guidance from multiple mentors				
No	394(48.40)	376.17	-4.38	< 0.001
Yes	420(51.60)	436.89		
Communicate candidly with supervisors to deepen mutual understanding				
No	194(23.83)	318.60	-7.18	< 0.001
Yes	620(76.17)	435.32		
Monthly academic communication with supervisors				
None	113(13.88)	368.64	16.25	0.001
< 3 times	303(37.22)	398.01		
< 5 times	176(21.62)	394.70		
≥ 5 times	222(27.27)	450.38		
Weekly one-on-one communication time with supervisors				
1–3 h	270(33.17)	379.05	12.24	0.007
4–6 h	357(43.86)	432.91		
> 7 h	88(10.81)	410.66		
1–3 h	99(12.16)	390.66		
Primary mode of communication with supervisors				
Face-to-face meetings	280(34.40)	436.63	13.72	0.008
Online platforms	310(38.08)	399.28		
Email	90(11.06)	362.97		
Phone calls or short messaging service	56(6.88)	366.77		
Online group or academic meetings	78(9.58)	416.23		
Supervisor-student relationships				
Good teacher/helpful friend	321(39.43)	466.91	64.77	< 0.001
Task-oriented	252(30.96)	399.85		
Ambivalent	86(10.57)	363.06		
Formalistic	132(16.22)	330.33		
Strained	23(2.83)	271.22		

#### Supervisor-student relationship classification

Based on the classification proposed by Chen [26], the supervisor-student relationship classification was divided into five categories, ranging from good to bad: good teacher/helpful friend, task-oriented, ambivalent, formalistic and strained relationships. Participants were asked to select the relationship type that best described their current relationship with their supervisor. Students and supervisors can collaborate more successfully to finish research and teaching tasks and form close emotional bonds when their interests align. The “good teacher/helpful friend” type indicates this. The “task-oriented” type demonstrates that both individuals are focused on completing the current task. Postgraduate students put forth a lot of effort to finish the tasks assigned by their supervisors and meet graduation criteria, but they rarely have emotional interaction. The “ambivalent” type refers to a relationship in which cooperation is desired but may be affected by differences in working styles, goals, or personalities. The “formalistic” type refers to a relationship in which supervisors and students mainly follow procedural requirements without active engagement. The “strained” type indicates a relationship characterized by serious tension or resentment, although the relationship may be maintained superficially because of concerns about “face” or practical interests [26].

#### Working Preference Inventory (WPI; student form)

Learning motivation was measured using the Working Preference Inventory (WPI; student form), developed by Amabile et al. [27] and later was translated and adapted into Chinese [28]. The 30-item instrument measures learning motivation using a 4-point scale ranging from 1 (strongly disagree) to 4 (strongly agree). The total score ranges from 30 to 120, with higher scores indicating stronger learning motivation. The subscales include intrinsic motivation, consisting of enjoyment and challenge, and extrinsic motivation, consisting of dependence on others’ evaluations, preference for simple tasks, concerns with competition, and pursuit of money or other tangible incentives. The Cronbach’s α in this study was 0.96.

#### Satisfaction with academic supervision

Overall satisfaction with academic supervision was assessed using a single-item self-rated 4-point ordinal measure: 4 = very satisfied, 3 = satisfied, 2 = dissatisfied, and 1 = very dissatisfied. Although satisfaction with academic supervision is generally considered a multidimensional construct encompassing aspects such as communication, guidance, feedback, and support [14], the present study aimed to capture students’ overall evaluation of their supervision experience rather than specific dimensions. Given the large-scale multicenter design of the survey and the need to minimize respondent burden, a single-item global measure was considered appropriate as a concise indicator of perceived satisfaction with academic supervision. Previous studies have shown that single-item global measures can provide acceptable validity when assessing general satisfaction or overall attitudes [26, 27]. Because the outcome was measured using a single item rather than a multi-item scale, internal consistency reliability (e.g., Cronbach’s α) was not applicable.

### Statistical analysis

Data analysis was performed using SPSS software version 26 (SPSS, Chicago, IL, USA). The normality of the continuous data was tested by the Kolmogorov-Smirnov test. As the continuous data were not normally distributed, non-parametric rank-based tests were used for univariable comparisons. Categorical variables were presented as frequencies and percentages. Because the satisfaction outcome was measured as an ordinal four-level variable, non-parametric rank-based tests were used for univariable comparisons in Table 1. Specifically, the Mann-Whitney *U* test was used for comparisons between two independent groups, and the test statistic was reported as *Z*. The Kruskal-Wallis *H* test was used for comparisons among three or more independent groups, and the test statistic was reported as *H*.

Variables with *p* < 0.05 in univariable analysis, together with the six dimensions of learning motivation, were entered into the ordinal logistic regression model with a cumulative logit link within the generalized linear model framework. To improve model parsimony and interpretability, variables that remained statistically significant in the multivariable analysis were retained in the final model. The six dimensions of learning motivation were entered as separate subscale scores. 

The results were reported as beta coefficients, standard errors, odds ratios (ORs) with 95% confidence intervals (CIs), Wald χ² values, and *p* values. Model fit was assessed using the likelihood ratio χ² test, Akaike information criterion (AIC), and Bayesian information criterion (BIC). The proportional odds assumption was assessed using the test of parallel lines, and multicollinearity was assessed using tolerance and variance inflation factor values. A two-sided *p* < 0.05 was considered statistically significant. The manuscript follows the Strengthening the Reporting of Observational Studies in Epidemiology (STROBE) guidelines.

### Ethical considerations

This study was conducted in accordance with the Declaration of Helsinki and was approved by the Ethics Committee of Hunan Normal University (Approval No. 2024 − 443). An information sheet was provided at the beginning of the online survey, which explained the study aims, voluntary nature of participation, data protection measures, and the right to withdraw at any time. Electronic informed consent was obtained from all participants before they accessed the questionnaire. Participants were required to indicate their consent by selecting “yes” before proceeding; those who selected “no” were not able to access the questionnaire. To minimize potential hierarchical influences, the survey was anonymous, universities and supervisors could not view individual responses, and the researchers had no supervisory/academic relationships with participants. Participation (or non-participation) would not affect personal interests.

## Results

### General information of participants

**Fig. 1:**
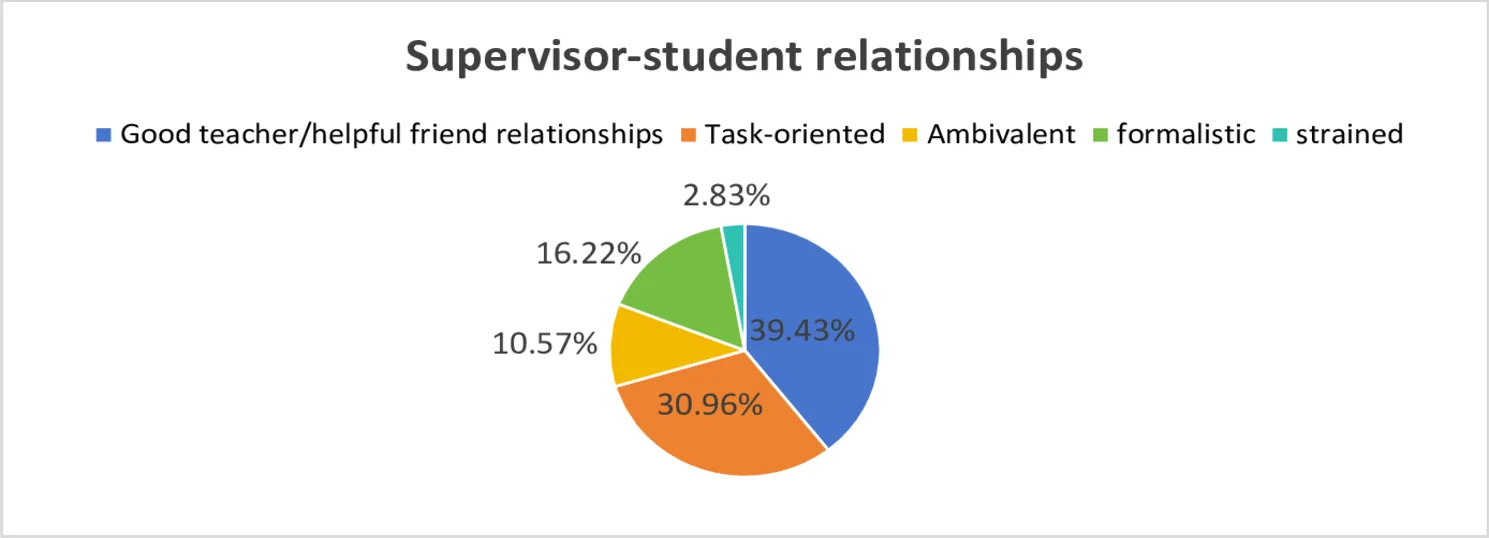
Distribution of supervisor-student relationship classifications (*N* = 814)

**Fig. 2:**
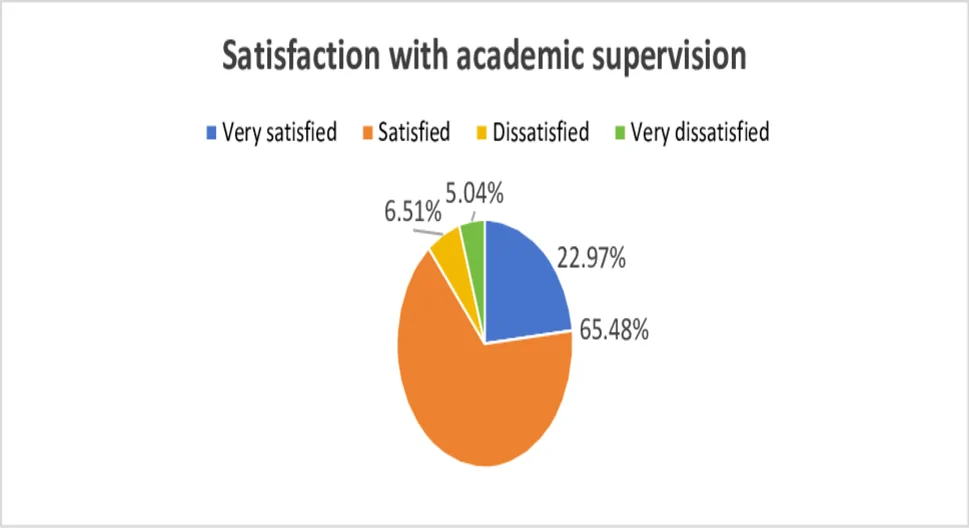
Distribution of satisfaction with academic supervision among nursing postgraduate students (*N* = 814)

A total of 814 nursing postgraduate students were included in this study. Most participants were female (80.00%), full-time students (90.54%), and enrolled in professional degree programs (65.11%). The most common supervisor-student relationship classification was “good teacher/helpful friend” (39.43%), followed by “task-oriented” (30.96%). Overall, 88.45% of participants reported being satisfied or very satisfied with academic supervision. The distributions of supervisor-student relationship classifications and satisfaction with academic supervision are shown in (Figs. 1 and 2.)

Univariable comparisons showed that satisfaction with academic supervision differed by undergraduate entrepreneurship experience, supervisor’s academic title, whether the supervisor was a Fellow of the American Academy of Nursing, whether the supervisor had no research projects, positive team atmosphere and support from senior members, regular group meetings and knowledge sharing, guidance from multiple mentors, communicate candidly with supervisors, monthly academic communication, weekly one-on-one communication time, primary mode of communication, and supervisor-student relationship. Detailed participant characteristics and univariable comparisons are presented in (Table 1).

### Factors associated with satisfaction with academic supervision

The final ordinal logistic regression model within the generalized linear model framework showed a significant overall fit, likelihood ratio χ² (degrees of freedom [df] = 9) = 308.490, *p* < 0.001. The AIC and BIC values were 908.015 and 964.438, respectively. Multicollinearity diagnostics showed that tolerance values ranged from 0.535 to 0.988 and variance inflation factor (VIF) values ranged from 1.012 to 1.868, indicating no serious multicollinearity. The proportional odds assumption was assessed using the test of parallel lines and was not violated, χ² (df = 18) = 27.833, *p* = 0.065.

In the final model, higher scores for challenge and pursuit of money or other tangible incentives were associated with higher odds of reporting greater satisfaction with academic supervision. The presence of a positive team atmosphere and support from senior members was also associated with higher satisfaction. In contrast, students without regular group meetings and knowledge sharing, and those without guidance from multiple mentors, had lower odds of reporting greater satisfaction. Compared with strained supervisor-student relationships, “good teacher/helpful friend” and “task-oriented” relationships were associated with higher satisfaction. The full model results are shown in (Table 2).

**Table 2:** Final ordinal logistic regression model of factors associated with satisfaction with academic supervision (*N* = 814)

Variable		Beta coefficient	Standard error	OR (95% CI)		Wald χ²		*p*	
Two dimensions of learning motivation									
Challenge	0.25		0.03		1.28 (1.20–1.36)		64.18		< 0.001
Pursuit of money or other tangible incentives	0.22		0.08		1.25 (1.07–1.46)		7.47		0.006
Positive team atmosphere and support from senior members									
No	Ref.								
Yes	0.59		0.25		1.81 (1.11–2.97)		5.56		0.018
Regular group meetings and knowledge sharing									
No	-0.88		0.21		0.41 (0.27–0.63)		17.19		< 0.001
Yes	Ref.								
Guidance from multiple mentors									
No	-0.45		0.16		0.64 (0.47–0.87)		8.15		0.004
Yes	Ref.								
Supervisor-student relationships									
Good teacher/helpful friend	2.01		0.47		7.42 (2.93–18.78)		17.91		< 0.001
Task-oriented	1.38		0.48		3.97 (1.56–10.06)		8.40		0.004
Ambivalent	0.79		0.51		2.20 (0.82–5.93)		2.44		0.118
Formalistic	0.79		0.49		2.19 (0.84–5.73)		2.56		0.108
Strained	Ref.								

Dependent variable: satisfaction with academic supervision scores, 4 = very satisfied, 3 = satisfied, 2 = dissatisfied, 1 = very dissatisfied

## Discussion

According to our study, 88.45% of nursing postgraduate students in China reported being satisfied or very satisfied with academic supervision, indicating a generally high level of satisfaction. This finding is consistent with studies from Australia, the United States, and several East and Southeast Asian countries, where more than 80% of nursing postgraduate students reported positive supervision experiences [29–31]. Although these studies differ in measurement and educational context, the findings suggest that satisfaction with academic supervision is generally high among nursing postgraduate students. Nevertheless, high overall satisfaction should not obscure variation in students’ supervision experiences or the factors associated with such variation.

From the perspective of the Input–Environment–Outcome framework [18], satisfaction with academic supervision can be viewed as an outcome shaped by both individual characteristics and the supervision environment. In this study, learning motivation represented an individual input factor, whereas team atmosphere, group meetings, guidance from multiple mentors, and supervisor-student relationships reflected environmental and process-related factors. This framework suggests that students’ evaluations of supervision are influenced by both who they are and how supervision is experienced.

Among the six dimensions of learning motivation, challenge and pursuit of money or other tangible incentives were associated with higher satisfaction. Students with a stronger challenge orientation may engage more actively with demanding academic tasks and feedback, making them more likely to view rigorous supervision positively [32–35]. In contrast, students who value tangible rewards may appreciate supervision that supports career development, research productivity, funding opportunities, and professional recognition [36–39]. These findings suggest that satisfaction may be higher when supervisory support aligns with students’ goals and expectations.

Supervision-related environmental factors were also associated with satisfaction. A positive team atmosphere and support from senior members may provide students with accessible role models, informal feedback, and emotional reassurance, supplementing formal supervision from faculty members [40–42]. Such environments may be particularly valuable when students feel reluctant to raise concerns directly with supervisors [43, 44]. These findings indicate that perceptions of supervision are shaped not only by supervisor-student interactions but also by the broader academic environment.

Regular group meetings and knowledge-sharing activities were likewise associated with higher satisfaction. These activities may provide opportunities for discussion, feedback, progress monitoring, and collaborative problem-solving while also strengthening students’ sense of belonging within the research team [45–47]. Guidance from multiple mentors was also linked to higher satisfaction, possibly because it provides access to diverse forms of expertise and support [48–51]. However, the effectiveness of multi-mentor arrangements likely depends on clear communication and role definition among mentors.

Supervisor-student relationships showed one of the strongest associations with satisfaction. Compared with strained relationships, relationships described as “good teacher/helpful friend” were associated with substantially higher satisfaction. Such relationships combine academic guidance with interpersonal support, mutual respect, and individualized attention, helping students feel valued and supported during their research training [52–54]. In Confucian-influenced educational settings, where hierarchical norms may sometimes limit open communication [55, 56], these findings highlight the value of supervisory approaches that balance academic authority with accessibility and trust.

Interestingly, task-oriented relationships were also associated with higher satisfaction than strained relationships. This suggests that positive supervision experiences do not depend solely on emotional closeness. For some students, clear expectations, structured guidance, well-defined goals, and appropriate interpersonal boundaries may be important indicators of effective supervision [57–59]. Rather than diminishing the importance of interpersonal support, this finding suggests that both relational and task-focused aspects of supervision can be associated with satisfaction.

Taken together, these findings indicate that satisfaction with academic supervision is associated with both students’ motivational orientations and characteristics of the supervision environment. The results emphasize the importance of aligning supervisory support with students’ needs and expectations. For postgraduate nursing education, initiatives aimed at supporting supervision quality should extend beyond individual supervisory practices and consider the broader academic environment. Supportive research team environments, regular group meetings and knowledge sharing, support from senior team members, and well-coordinated multi-mentor arrangements may all contribute to more positive supervision experiences. In Confucian heritage contexts such as China, South Korea, and Japan, approaches that combine clear academic expectations with supportive interpersonal engagement may be particularly beneficial. Future initiatives should therefore address both the relational and organizational dimensions of supervision rather than focusing solely on individual supervisors.

## Limitations

This study has several limitations. First, the cross-sectional design limits causal inference. Future longitudinal or intervention studies are needed to further examine potential causal pathways.

Second, although universities were selected using a stratified proportional cluster strategy, participants within each university were recruited through voluntary online participation rather than individual-level random sampling. Therefore, volunteer bias and self-selection bias may have occurred, which may limit the generalizability of the findings. Future studies should consider probability-based sampling or broader multicenter recruitment to improve sample representativeness.

Third, all data were collected using self-reported online questionnaires. Self-report bias and social desirability bias cannot be excluded, although anonymity was used to reduce this risk. In addition, because both independent variables and the outcome were collected from the same questionnaire at the same time, common method bias may have occurred. Future studies could combine self-reported data with supervisor evaluations, institutional records, or qualitative interviews to provide a more comprehensive assessment.

Fourth, the measurement approach had limitations. Satisfaction with academic supervision was assessed using a single-item 4-point ordinal measure, which captured global satisfaction but could not reflect specific dimensions such as communication, feedback, academic guidance, and psychosocial support. Several supervision-related variables were also measured using study-specific single items that did not undergo formal psychometric validation. Future studies should develop or apply validated multi-item instruments to assess satisfaction with academic supervision and supervision-related experiences more comprehensively.

## Conclusions

Nursing postgraduate students reported high levels of satisfaction with academic supervision. Satisfaction was associated with the challenge and pursuit of money or other tangible incentives dimensions of learning motivation, positive team atmosphere and support from senior members, regular group meetings and knowledge sharing, guidance from multiple mentors, and supervisor-student relationships. Supervisors may consider aligning their guidance with students’ learning motivations, while universities and colleges may support structured mentorship groups, positive team climates, and constructive supervisor-student relationships to strengthen postgraduate nursing education.

## Data Availability

The datasets used and/or analysed during the current study are available from the corresponding author on reasonable request.
